# Safety of TNF-α inhibitors during IBD pregnancy: a systematic review

**DOI:** 10.1186/1741-7015-11-174

**Published:** 2013-07-31

**Authors:** Ole Haagen Nielsen, Edward V Loftus Jr, Tine Jess

**Affiliations:** 1 Department of Gastroenterology, Medical Section, Herlev Hospital, University of Copenhagen, Copenhagen, Denmark; 2 Division of Gastroenterology & Hepatology, Mayo Clinic, Rochester, MN, USA; 3 Department of Epidemiology Research, Statens Serum Institute, National Health Surveillance and Research, Copenhagen, Denmark

**Keywords:** Biologics, Crohn’s disease, IBD, Lactation, Pregnancy, Ulcerative colitis

## Abstract

**Background:**

Tumor necrosis factor (TNF)-α inhibitors are increasingly being used in inflammatory bowel disease (IBD). Because this chronic intestinal disorder often affects women of fertile age, it is essential to assess the effect of biologics on pregnancy outcome.

**Methods:**

We performed a systematic review of the English-language literature to investigate if treatment with TNF-α blockers during pregnancy in women with IBD increases the risk of spontaneous abortions, preterm delivery, stillbirth, low birth weight, congenital malformations, or risk of infections in the offspring. Of 552 articles and abstracts reviewed, 58 articles or abstracts with unique content were identified and included in this systematic review. However, most presentations were case reports or case series supplied by a limited number of observational studies. No randomized controlled studies were available.

**Results:**

TNF-α inhibitors do not seem to affect either outcome of pregnancy in mothers with IBD, or the outcome in the offspring (congenital malformations and immunosuppression). Further, recent data have not identified any increased risk of infections in the first year of life in the offspring of mothers who received biologics, even in combination with immunomodulators (thiopurines).

**Conclusions:**

From the present systematic review, no association was found between administration of TNF inhibitors for IBD during pregnancy and adverse pregnancy outcome or congenital abnormalities. Further, no increased relative risk of infections has been reported in the first year of life in offspring of mothers who received biologics. Biologics should be discontinued during pregnancy solely if the IBD is in remission using the same stopping criteria as for patients with IBD in general, as uncontrolled activity of IBD may expose the mother and child to a risk greater than those only potentially coming from the use of TNF-α inhibitors. In such cases, inoculation of the offspring with live vaccines is contraindicated until the biologic agent is no longer detectable in the child’s circulation.

## Background

Biologics are effective and increasingly used in the treatment of inflammatory bowel disease (IBD), of which Crohn’s disease (CD) and ulcerative colitis (UC) are the two main types. As the peak incidence of IBD overlaps with the prime reproductive years, information on the effects of biologics on pregnancy outcome (including fetal harm) is essential in order to give appropriate advice to women of childbearing age who require treatment for IBD.

In general, women with IBD are advised to conceive at quiescent stages of their disease, as this reduces the risk of obstetric complications (for example, preterm delivery and low birth weight) [1-5]. Effective control of disease activity using a wide range of medications, including biologics, is vitally important during pregnancy [6,7].

The most commonly used biologics in IBD are infliximab (IFX), a chimeric IgG1 monoclonal antibody; adalimumab (ADA), a human monoclonal IgG1 antibody; and certolizumab pegol (CZP), a pegylated Fab fragment of a humanized IgG4 isotype monoclonal antibody [8]. Recently, golimumab (GLM), also a human monoclonal IgG1 antibody, has been added to the list. These four biologic agents are all classified as ‘category B’ drugs by the US Food and Drugs Administration (FDA) [9], meaning that animal reproduction studies have not shown any risk to the fetus, but there are no adequate and well-controlled studies in pregnant women.

In the clinical scenarios of a woman with IBD on biologic therapy becoming pregnant, or when biologic treatment is being considered in a pregnant woman with active IBD, decisions are made on a case-by-case basis because the short-term and long-term implications of exposure to biologic agents have not yet been investigated systematically. Although no comparative effectiveness data exist to support the strategy, it has been common practice at many IBD centers to continue biologics through the second trimester of pregnancy [10], as the transplacental transfer of IgG begins around week 20 and increases thereafter, especially during the third trimester [11]. In accordance with this practice, IFX and ADA are discontinued around week 30 of pregnancy [12], and in patients with active IBD, it has been suggested to bridge therapy with glucocorticoids to control disease activity until delivery [13-16]. This approach has the benefit of minimizing the break from treatment with biologic agents and perhaps decreasing the risk of immunogenicity [8] (that is, the risk of hypersensitivity reactions or loss of response once the biologic is reintroduced post-partum). However, CZP may differ from the other tumor necrosis factor (TNF)-α blockers used in IBD, as it is a Fab fragment of a monoclonal antibody, which is not transported across the placenta and therefore, it may not be necessary to discontinue this drug in the third trimester [17].

However, at present there are no definitive conclusions concerning what could be one of the most important therapeutic decision-making settings from the viewpoint of risk-benefit analysis. Thus, the decision whether to use biologics during pregnancy might be regarded as an example in which it is considered most prudent to err on the side of caution, especially as it is still unknown what levels in the serum of TNF inhibitors used to treat a mother with IBD will be safe or harmful for the fetus or newborn.

The aim of this study was to perform a systematic review of the available literature on the risk of adverse birth outcomes related to IBD after maternal exposure to IFX, ADA, or CZP, in order to improve evidence-based informed choice and clinical decision-making during pregnancy, especially in the third trimester. Data about use of GLM in IBD pregnancies are currently unavailable, as it has only recently been approved for UC.

## Methods

### Ethics approval

This study was an analysis of published data, which did not require ethics committee approval.

### Search strategy

A systematic review was performed according to the guidelines established by the Meta-analysis of Observational Studies and Epidemiology Group (MOOSE criteria) [18]. The databases searched (January 1998 to May 2013) were MedLine, EMBASE, the Cochrane Library, and the homepages of the FDA and the European Medicines Agency (EMA), using the combinations of the following Medical Subject Heading (MeSH) search terms: (inflammatory bowel disease OR inflammatory bowel diseases OR Crohn’s disease OR ulcerative colitis) AND biologics OR biologic products OR infliximab OR adalimumab OR golimumab OR certolizumab. This was again combined with either pregnancy OR newborn OR lactation OR breast feeding OR infant. No authors were contacted.

### Selection criteria

**Figure 1:**
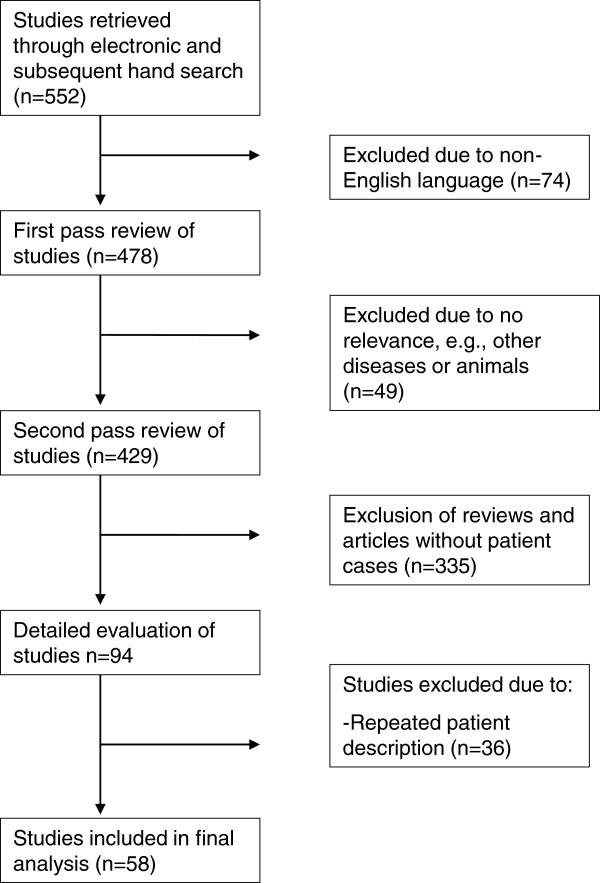
Flowchart of the study screening process.

We wanted to include all randomized controlled trials, observational studies, case series, and case reports evaluating pregnancy outcomes of women with IBD treated with TNF-α inhibitors (IFX, ADA, GLM, or CZP) during any trimester of pregnancy, including the 90 day periconceptional period. Outcome assessment included miscarriages (spontaneous abortions), preterm delivery, stillbirth, low birth weight, and/or congenital malformations, and if data were available, it also included assessment of infections in the offspring. Only abstracts or articles published in English were considered. Figure 1 shows the flowchart of the study screening process.

### Data extraction

Two authors (OHN and TJ) independently identified candidate articles from the results of the initial search, based on the title and abstract. Subsequently, both authors independently reviewed the full text of candidate articles to identify interventions and assess study quality. In cases of any discrepancies between the independent searchers, these were resolved in consensus with the third author (EVL).

The reference lists of relevant articles were hand-searched to identify additional studies. Further, abstracts from the meetings of American Gastroenterological Association (Digestive Disease Week), American College of Gastroenterology, and the European Crohn’s and Colitis Organisation from 1998 to May 2013 were searched manually for relevant abstracts based on the aforementioned search terms.

### Summarization of data

The search yielded a total of 58 studies (36 articles and 22 abstracts) that met the inclusion criteria (Table 1). The studies comprised 33 case reports, 21 case series, and 4 prospective studies with or without control groups. No controlled randomized studies were identified. The total number of pregnant women with IBD who were exposed to anti-TNF agents was at least 1,533 (one study with 289 pregnancies included other diseases and did not specify the number of women with IBD [19]). The bias of the present study was that only non-English studies were excluded.

**Table 1:** **Published studies on the use of TNF**-**α blockers in IBD pregnancies**

**Author**	**Year**	**Anti**-**TNF**	**Stage of pregnancy**	**Study type**	**Mode of delivery**	**Outcome of pregnancies**
James [25]	2001	IFX	P(T2)	Case report	Unknown	Normal, healthy, full term
Srinivasan [21]	2001	IFX	PC/P(T1)	Case report	Unknown	Premature, preterm, LBW. Adverse outcome: intracerebral and intrapulmonary bleeding followed by death 3 days later (metronidazole + azathioprine + 5-ASA was also administered in pregnancy for treatment of flare and fistulae)
Bank *et al*. [24]	2002	IFX	P(T1-T2)	Case report	Unknown	Normal, healthy, full term (correspondence with authors)
Burt *et al*. [27]	2003	IFX	PC	Case report	C-section	Normal, healthy, preterm (36 weeks)
Katz *et al*. [49]	2004	IFX	53/96 PC; 58/96 P(T1); 6/96 unknown	Case series from Infliximab Safety Database (82 CD, one UC, 10 RA, 3 unknown); 96 women, 72 births	Unknown	64/96 were live births (1 preterm death at 24 week, 1 full-term with tetralogy of Fallot, 1 perinatal sepsis, 1 intestinal malrotation, 1 developmental delay); 4 pregnancies ended with twins. 14/96 were miscarriages, 18/96 were therapeutic terminations
Mahadevan *et al.*[32]	2005	IFX	9/10 PC; 8/10 P(T2); 8/10 P(T3); 7/10 PP	Case series (n = 10 women)	8/10 C-section, 2/10 Vaginal	3/10 were preterm, 1/10 were LBW, 2/10 were neonatal illnesses, which resolved. All normal at follow-up
Tursi [34]	2006	IFX	PC/P(T1-T3)/PP	Case report	Unknown	Normal, healthy, preterm (36 weeks)
Xirouchakis *et al*. [64]	2006	IFX	PC/P(T1)	Case report	Vaginal	Normal, healthy, full term
Vasiliauskas *et al*. [16]	2006	IFX (10 mg/kg body weight)	PC/P(T1-T3)	Case report	Vaginal	Normal, healthy, full term
Mahadevan *et al*. [28]	2007	IFX	P(T1-T3)	Case series (n = 5)	4 vaginal/1 C-section	Normal, healthy, full term
Malgarinos *et al*. [29]	2007	IFX	PC/P(T1-T3)	Case report	Unknown	Normal, healthy, preterm
Angelucci *et al*. [26]	2008	IFX	P(T1)	Case report	C-section	Normal, healthy, full term
Palmer *et al*. [20]	2008	IFX	P(T2-T3)	Case report	Unknown	Intra-uterine fetal death due to placental abruption or intra-uterine infection
Stengel *et al*. [33]	2008	IFX	PC/P(T1-T3)	Case report	C-section	Normal, healthy, full term
Kane *et al*. [68]	2009	IFX	2 PC/P(T1-T3), 1 P(T2)	Case series (3 women)	2 vaginal, 1 C-section	3 normal, healthy, full term
Hou *et al*. [14]	2009	IFX	PC/p(T1-T3)	Case report	Vaginal	Normal, healthy, full term
Arai *et al*. [23]	2010	IFX	P(T2-T3)	Case report	Unknown	Normal, healthy, full term
Epping *et al*. [66]	2010	IFX	PC/P(T1-T3)	Case report	Unknown	Normal, healthy, full term
Angelucci *et al*. [65]	2010	IFX	PC/P(T1)	Case report	C-section	Normal, healthy, preterm (31 weeks)
Correia *et al*. [30]	2010	IFX	PC/P(T1-T3)	2 case reports	C-section	Normal, healthy; 1 preterm (31 weeks)
Molnár *et al*. [47]	2010	IFX	PC/P(T1-T3)	Case report	Unknown	Normal, healthy, full term
Cheent *et al*. [63]	2010	IFX (10 mg/kg body weight)	PC/P(T1-T3)	Case report	Vaginal	Healthy, full term, no malformations
Aratari *et al*. [44]	2011	IFX	P(T2)	Case report	C-section	Healthy, preterm (37 weeks)
Abdul Wahab *et al*. [40]	2011	IFX	P(T2-T3)	Case report	Unknown	Normal, healthy, full term
Kozeluhova [41]	2011	IFX	PC/P(T1-T3)	Case report	Unknown	Normal, healthy, full term
Chaparro *et al*. [45]	2011	IFX	PC/P(T1-T3)	Case report	C-section	Normal, healthy, full term
Naganuma *et al*. [67]	2011	IFX	PC/P(T1-3)	Case series (3 women)	Unknown	All healthy, full term, 1 low birth weight, no malformations
Steenholdt *et al*. [22]	2012	IFX	PC/P(T1-T2)	Case report	Unknown	Normal, healthy, full term
Arguelles-Arias *et al*. [42]	2012	IFX	PC/P(T1-T3)	Case series (12 women)	8 vaginal, 4 C-sections	12 healthy children
Lichtenstein *et al*. [36]	2012	IFX	142 reported pregnancies	Case series (analysis of data from TREAT registry)	Unknown	The vast majority of babies (92.4%) were healthy with no defects or other adverse events
Snoeckx *et al*. [38]	2013	IFX	PC/P(T1-T3)	Case series (556 pregnancies)	Unknown	There were 58 miscarriages, 38 elective abortions, 7 stillbirths, and 453 live births
Vesga *et al*. [80]	2005	ADA	PC/P(T1-T3)/PP	Case report	C-section	Normal, healthy, full term
Coburn *et al*. [77]	2006	ADA	P(T2; T3)	Case report	Unknown	Normal, healthy, full term
Mishkin *et al*. [79]	2006	ADA	PC/P(T1-T3)	Case report	Vaginal	Normal, healthy, full term
Chambers *et al*. [75]	2006	ADA	PC/P(T1)	Case series (5 women)	Unknown	All full-term live births. No malformations
Bosworth *et al*. [71]	2007	ADA	PC/P(T1-T3)	Case report	C-section	Normal, healthy, full term
Johnson *et al*. [72]	2009	ADA	PC/P(T1-T3)	Case-controlled study 34 women and 45 controls (OTIS)	Unknown	29 live-born (1 with undescended testicle + 1 with microencephaly); 5 spontaneous abortions. Two major structural defects in control group
Jürgens *et al*. [78]	2010	ADA	PC/P(T1)	Case report	Vaginal	Healthy, full term
Ben-Horin *et al*. [74]	2010	ADA	PC/P(T1-3)	Case report	Vaginal	Healthy, full term
Abdul Wahab *et al*. [73]	2011	ADA	PC/P(T1-T3)	Case report	C-section	Twins, normal, healthy, full term
Mahadevan *et al*. [76]	2011	ADA	PC/P(T1-T3)	Case series (5 women)	2 vaginal 3 C-sections	Healthy (one infant developed pulmonary edema, but recovered fully)
Kane *et al*. [31]	2009	CZP	Unknown (UCB data on file)	Case series (16 women)	Unknown	4 healthy, full term; 1 small for gestational age; and 2 lost to follow-up. 8 induced abortions amd 1 spontaneous abortion
Mahadevan *et al*. [85]	2009	CZP	P(T2; T3)/PP	Case report	Unknown	Healthy, full term, no malformations
Oussalah *et al*. [86]	2009	CZP	PC/P(T1; T3)	Case report	Vaginal	Normal, healthy, full term. Adverse outcome in 1 case: bleeding 7 days post-partum, secondary to cotyledon retention
Steinberg *et al*. [84]	2010	CZP	P(T2-T3)	Case report	C-section	Healthy, full term, no malformations
Mahadevan *et al*. [87]	2012	CZP	PC/P(T1-T3)	Case series (139 pregnancies including 17 with rheumatoid arthritis)	Unknown	21 miscarriages, 15 elective abortions, and 103 live births (2 with congenital abnormalities: 1 with mild unilateral hydronephrosis and 1 with vesicouretic reflux)
Slama *et al*. [19]	2010	IFX/ADA	PC/P(T1-T3)	Case series (289 pregnancies including other diseases than IBD)	Unknown	No increased risk of adverse pregnancy outcome compared with unexposed IBD pregnancies
Dunne *et al*. [46]	2011	IFX/ADA	PC/P(T1-T3)	15 case series (15 women)	Unknown	9 terminated pregnancies at study end. All live births were healthy, with two sets of twins
Schnitzler *et al*. [54]	2011	IFX/ADA	PC and/or P(T1) and/or P(T2)	Case-controlled study (35 women with 42 pregnancies (35 IFX + 7 ADA) versus 101 pregnancies before IBD/treatment and 56 matched pregnancies in healthy controls)	Unknown	10 abortions (1 trisomy 18, 1 elective), 1 stillbirth (umbilical strangulation), 32 live births: 8/32 premature, 6/32 low birth weight. All children were born healthy but one child with necrotizing enterocolitis died at 13 days of age (mother required treatment for severe asthma and CD during T3)
Machková *et al*. [43]	2012	IFX/ADA	PC/P(T1-T3)	Case series (33 pregnancies in 31 women)	Unknown	5/33 pregnancies ongoing. 24/28 healthy children (4/24 preterm), 3/24 spontaneous abortions, and 1/24 provoked abortion
Habal *et al*. [37]	2013	IFX/ADA	PC/P(T1-T3)	Case series (29 pregnancies in 21 women)	Unknown	27 normal, healthy, full term; 2 miscarriages
Traussnigg *et al*. [39]	2013	IFX/ADA	PC/P(T1-T3)	Case series (14 pregnancies in 13 women)	Unknown	13 normal, healthy, full term; 1 miscarriage
Zelinkova Z *et al*. [12]	2013	IFX/ADA	PC/P(T1-T3) (T1-T2 ADA only)	Case series (31 pregnancies in 28 women)	Unknown	28 live births (27 normal, healthy, full term; 1 child born to a mother treated with concomitant methotrexate periconceptually had polydactyly); 3 miscarriages
Casanova *et al*. [48]	2011	IFX/ADA/CZP	PC/P(T1-T3)	Case-controlled retrospective study (30 pregnancies in treated mothers versus 110 unexposed IBD pregnancies)	Unknown	Prevalence of unfavorable delivery outcome (spontaneous abortion, preterm delivery, low birth weight and malformations) were higher in the control group
Seirafi *et al*. [52]	2011	IFX/ADA/CZP	PC/P(T1-T3)	Case series (85 women)	Unknown	6/85 were miscarriages, 47/85 were live births (30/85 ongoing), 8/49 were preterm, 3/49 were deaths (two *in utero*/one very premature),7/46 live births had neonatal complications (1 infection, 3 respiratory distress syndrome, 6 LBW <2500 g, 1 HELLP syndrome
Mahadevan *et al*. [51]	2012	IFX/ADA/CZP	PC/P(T1-T3)	Case-controlled study (291 women without, and 79 with immunomodulators)	Unknown	No increased risk apart from higher risk of spontaneous abortion and C-section in group treated with biologics alone, and higher risk of preterm birth and infections int offspring at 12 months in combined thiopurine group verus biologics-alone group or unexposed IBD pregnancies
Casanova *et al*. [35]	2013	IFX/ADA/CZP	PC/P(T1-T3)	Case series (66 pregnancies)	Unknown	55 normal, healthy full term;, 4 preterm; 6 miscarriages; 1 congenital abnormality (several cardiac malformations)
Mahadevan *et al*. [17]	2013	IFX/ADA/CZP	PC/P(T1-T3)	Case series (31 women)	15 vaginal, 16 C-section	All 33 children (2 sets of twins) were normal, healthy, full term

Abbreviations: *5-ASA* 5-aminosalicylic acid, *ADA* adalimumab; *C-section* cesarean section, *CZP* certolizumab pegol, *HELLP* a syndrome characterized by hemolysis, elevated liver enzymes, and low platelet count (that is, a variant/complication of pre-eclampsia), *IBD* inflammatory bowel disease, *IFX* infliximab, *LBW* low birth weight, *PC* periconceptual (that is, use within 90 days before conception), *PP* post-partum, P(T1-T3) (pregnancy with trimester where biologics were reported to be administered), *MTX* methotrexate, *TNF* tumor necrosis factor, *TREAT* Therapy Resource Evaluation Assessment Tool.

## Results

### Infliximab

In the early days of biologic treatment for IBD, IFX was reported to be associated with adverse pregnancy outcomes in some case reports [20,21], but not in others [22-27], and in case series where IFX was administered throughout pregnancy to maintain remission in IBD, no harm to the fetus/child was reported [16,19,28-34]. Furthermore, larger and subsequent studies did not report any increased risk for adverse events compared with unexposed IBD pregnancies [12,17,35-49]. In a study on pregnancies exposed to IFX, Zelinkova *et al*. observed one adverse event (polydactyly), actually reported on two occasions [12,50]. In this particular case, the mother of the infant had also received methotrexate (MTX) 2 months prior to conception [12,50]. MTX is a drug classified as ‘X’ by the FDA; that is, a drug for which studies in animals or humans have shown fetal abnormalities and/or for which there is positive evidence of human fetal risk, based on adverse reaction data from investigational or marketing experience, and for which the risks involved in use of the drug in pregnant women clearly outweigh the potential benefits [9]. However, polydactyly is an uncommon disorder; it is not usually seen as the only complication to MTX treatment, but is more often present together with other abnormalities [50].

The first series studying intentional IFX administration throughout pregnancy examined outcomes in 10 women with active CD, and all ended in live births [32]. Of these 10 births, there were 3 cases of premature birth and 1 case with low birth weight, findings that were not unexpected in women with CD that was sufficiently severe to require IFX treatment. No congenital abnormalities were observed 6 months post-partum [32].

The four largest studies providing data on the safety of IFX in an IBD population are from the Therapy Resource Evaluation Assessment Tool (TREAT) registry [36], a pregnancy safety database maintained by the manufacturer of IFX [49], the Pregnancy in Inflammatory Bowel Disease and Neonatal Outcomes (PIANO) registry [51] and the Groupe d’Etude Thérapeutique des Affections Inflammatoires du Tube Digestif (GETAID) collaboration [52].

The TREAT registry is a prospective study of North American patients with CD. As of February 2010, there were 6,273 patients in the registry, and 142 pregnancies with IFX exposure were reported. The risk of fetal malformations was no higher than in the general population [36], and a more detailed analysis of pregnancies in the TREAT registry is under way [36].

The IFX Safety Database is a retrospective data-collection instrument set up by the manufacturer of IFX (Centocor, now Janssen Biotech) to study women with rheumatoid arthritis or CD exposed to IFX before or during pregnancy. There were 96 women with direct exposure to IFX who gave birth to 100 children; however, in most case, IFX treatment was discontinued in the first trimester of pregnancy. The rates of adverse outcomes were not different from that expected in the general population [49].

Preliminary data from the US national prospective PIANO registry has recently been disclosed [53]. A total of 1,232 women with IBD were enrolled as of April 1, 2013, of whom 357 women received biologics, including during the third trimester, and an additional 109 women received a combination of thiopurines and biologics [53]. Adverse pregnancy outcomes such as spontaneous abortions, preterm birth, low birth weight, and congenital abnormalities were evaluated. A slightly higher relative risk (RR) for preterm birth was found for women on combination therapy (2.4; 95% confidence interval (CI) 1.3 to 4.3), which was, however, not found for those on biologics only (0.8; 95% CI, 0.5 to 1.3). No elevation in the risk of spontaneous abortion, low birth weight, or congenital abnormalities was observed for biologics, either alone or in combination with thiopurines. Earlier reports (May 2012) had indicated that there was a higher rate of infections in the offspring [51], but the RR at month 12, adjusted for premature birth, was shown in April 2013 to be 0.9 (95% CI 0.7 to 1.1) for biologics alone, and 1.0 (95% CI 0.7 to 1.3) for those on combined therapy. When results for CZP were excluded, the values were 0.9 (95% CI 0.8 to 1.2) and 1.0 (95% CI 0.7 to 1.3), respectively [53]. In addition, breast feeding was not associated with any risk of infection or impairment of height or weight. The limitation on the preliminary data was that not all infants had reached the age of 12 months, and the observation will continue until the age of 4 years for all of the offspring [53].

The French GETAID consortium prospectively recorded pregnant women with IBD during a 2 year period (2009 to 2010), and preliminary data analysis of 127 pregnancies in 120 women was reported in 2010 [52]. Of these 120 women, 54 women had received biologic therapy for their IBD. Birth outcomes of the women on biologics did not differ from those in the unexposed IBD population, suggesting an absence of any excess risk linked to anti-TNF-α therapy.

Schnitzler *et al*. [54] conducted an observational study of 35 pregnancies under direct exposure to IFX until week 20 of pregnancy, 53 pregnancies with indirect exposure to IFX (women treated with IFX prior to pregnancy), 7 pregnancies with direct exposure to ADA, and 56 matched pregnancies in healthy women. Several patients experienced a flare of disease in the third trimester. However, exposure to anti-TNF-α preparations was not associated with a higher incidence of adverse pregnancy outcomes [54]. The paper concluded that pregnancy outcomes after exposure to TNF inhibitors did not differ from those of women with IBD who were not exposed to anti-TNF treatment, but were worse than in healthy women [54]. Further, a literature review including women with rheumatic disorders or CD with direct exposure to IFX at any time during pregnancy did not find any increase in undesirable pregnancy or fetal outcome [55].

However, in contrast to studies providing evidence of the safety of anti-TNF-α preparations during pregnancy, a review of more than 120,000 adverse events voluntarily reported to the FDA after drug exposure revealed 61 congenital abnormalities in 41 children of 40 mothers exposed to TNF-α blockers for various indications, including IBD [56]. Of these, 22 mothers were exposed to etanercept (a biologic agent not efficacious for IBD [57]), and 19 were exposed to IFX. Of the 41 children, 24 (59%) had abnormalities thought to be part of the VACTERL (vertebral abnormalities, anal atresia, cardiac defects, tracheoesophageal, renal and limb abnormalities) spectrum. The findings led the authors to conclude that the use of TNF-α blockers should be avoided during pregnancy. However, this study was subsequently criticized for significant methodological flaws [58,59], including selection bias, lack of a control group, and the fact that only one of the infants (the etanercept group only) had a clustering of three or more abnormalities included in the VACTERL spectrum [60]. Furthermore, the review did not take into account the total number of women exposed to anti-TNF-α agents or any confounding by severity of the underlying disease. Finally, the most common defects reported in this review were cardiac defects, which are observed in the general population as well [58,59].

Like other IgG antibodies, IFX is transferred across the placenta with the help of an Fc receptor neonatal (FcRn) molecule responsible for the transfer of immunoglobulins from the mother to the neonate [11]. The transfer occurs partly in the second, and mainly in the third trimester [31]. This finding has led many clinicians to discontinue IFX in the third trimester when the highest level of transfer occurs [61], in the belief that detectable serum IFX in the neonate might lead to clinically relevant immunosuppression. Moreover, the use of biologics in the second and third trimesters has been questioned [12]. However, Zelinkova *et al*. [12,50] showed that even though two patients discontinued IFX treatment as early as in week 26 of pregnancy, the infants still had detectable amounts of IFX in their serum samples after delivery. Low levels of IFX have been detected up to 6 months post-partum in the serum of infants whose mothers have received this drug up to delivery [16]. IFX was undetectable in the infant at delivery only in one case, where the mother discontinued drug therapy at week 21, suggesting that discontinuation of drug therapy might be considered earlier than the beginning of the third trimester [50].

Because of this presence of IFX in the circulation after delivery, the use of live-virus vaccines (for example, varicella, measles, mumps, rubella, rotavirus, intranasal influenza, and bacillus Calmette-Guérin; BCG) is contraindicated in patients receiving biologic therapy and their children [62]. A case report described a fatal incident of an infant who was exposed to IFX during gestation and who received a BCG vaccine at 3 months; this infant died of disseminated BCG infection [63]. Thus, the most recent recommendation from the World Congress of Gastroenterology consensus statement on vaccinations in infants exposed to biologic therapy *in utero* recommend delay of all live-virus vaccines until after biologic molecules are no longer detectable in the child’s blood [62].

In this systematic review, identifying 17 case reports related to IFX [14,16,20,22,23,29,30,33],[34,40,41,45,47,63-66], 13 case series [12,17,19,28,32,37-39,42,43],[46,67,68], 2 uncontrolled cohort studies [19,36, and 2 controlled cohort studies [48,69] (Table 2), we found the prevalence of pregnancy complications, including preterm delivery, stillbirth, low birth weight, miscarriages, or congenital malformations in children exposed to IFX throughout pregnancy is limited, even after exposure to biologics throughout the third trimester. However, the use of IFX up to week 30 of gestation results in fetal intra-uterine exposure to high IFX levels (up to three-fold higher than in the maternal peripheral blood), which may raise concerns about the long-term effects of IFX on these children, including effects on their immune system [50].

**Table 2:** **Studies reporting exact numbers**, **odds ratios**, **or relative risks for various birth outcomes in women with IBD exposed to TNF**-**α blockers compared with unexposed controls with IBD**

**Author**	**Patients with IBD exposed to TNF**-**α blockers**	**Controls**	**Spontaneous abortions or stillbirths**	**Preterm delivery**	**Low birth weight**	**Abnormalities**/**major birth defects**/**malformations**	**Infections during the first 12 months of life**
Johnson *et al*. [72]	34^a^	55^b^	5/34 versus 3/55; OR 2.99 (95% CI 0.67 to 13.41)	−	−	2/34 versus 2/53; OR 1.59 (0.21 to 11.9)	−
Schnitzler *et al*. [54]	42	78	9/42 versus 12/78; OR 1.5 (0.57 to 3.92)	8/32 versus 8/65; OR 2.38 (0.80 to 7.06)	6/32 versus 9/65; OR 1.44 (0.46 to 4.46)	1/42 versus 1/78; OR 1.87 (0.11 to 30.8)	−
Casanova *et al*. [48]	202^c^	110	20/202 versus 19/110; OR 0.53 (0.27 to 1.04)	8/202 versus 13/110; OR 0.31 (0.12 to 0.77)	12/202 versus 11/110; OR 0.57 (0.24 to 1.33)	2/202 versus 0/110; (no OR or CI)	−
Mahadevan *et al*. [51]	79^c^	291/326	RR 1.29 (0.79 to 2.11); Biologics alone:; RR 2.56 (1.07 to 4.12)	RR 1.83 (1.01 to 3.31); Biologics alone:; RR 0.89 (0.54 to 1.47)	−	−	RR 1.35 (1.01-1.80); Biologics alone:; RR 0.89 (0.70-1.12)

Abbreviations: *CI* confidence interval, *IBD* inflammatory bowel disease, *OR* odds ratio, *RR* relative risk, *TNF* tumor necrosis factor.

^a^Patients with Crohn’s disease, psoriasis, rheumatoid arthritis, and other forms of arthritis.

^b^Controls were unexposed patients with rheumatoid arthritis.

^c^TNF-α blockers and/or thiopurines or other immunomodulators.

### Adalimumab

The clinical data for the safety of ADA during pregnancy in women with IBD are more limited than for IFX, but in animals, it appears that ADA does not increase obstetric risks and has no teratogenic effects [70]. In humans, data on ADA and pregnancy primarily concerns patients with diseases other than IBD, such as rheumatoid arthritis and psoriasis.

For IBD, 21 case reports and series with more than 300 children exposed showed no increased risk of adverse pregnancy outcome or congenital malformations associated with ADA treatment during pregnancy compared with pregnancies in unexposed women with IBD [12,17,19,35,37,39,43,46],[48,51,52,54,71-80], even if ADA was administered in the third trimester [17,19,35,37,39,43,46,48],[51,52,71-74,76,77,79,80]. The Organization of Teratology Information Specialists reported on a group of 34 women treated with ADA for various indications in a prospective study, and another 133 ADA-exposed women in a case series. There was no difference in preterm deliveries, stillbirths, spontaneous abortions or congenital malformations in the group treated with ADA compared with either the general population or a control group with the same disease but not exposed to ADA [62,81]. In line with these observations, Schnitzler *et al*. did not find a higher incidence of adverse pregnancy outcomes in seven women treated with ADA compared with unexposed women with IBD, although the power of that study was limited [54].

An ongoing cohort study evaluating ADA in pregnancy compared 80 live offspring born to 94 ADA-exposed women with CD, 53 live offspring of 58 women with CD not exposed to ADA, and 78 of 87 women from a non-diseased comparison group. The frequency of congenital abnormality in the three groups was 9.6%, 5.4%, and 5.0%, respectively [72] (*P*>0.05 between all groups).

Like IFX, ADA is transferred across the placenta in the third trimester of pregnancy [31,76], and accordingly, it has been recommended at many IBD centers that ADA should be discontinued in the third trimester (that is, week 30), even though data, as mentioned before, do not support this theory [82].

Osting *et al*. carried out a review of TNF-α blockers (ADA and eternacept only) administered during pregnancy from 1999 to 2009 using the Organization of Tetratology Information Specialists (OTIS) prospective registry, and concluded that 7 to 10% of children had congenital abnormalities [83]. However, the underlying disease activity might be a confounding factor with greater effect on the risk of congenital abnormalities than the biologic treatment *per se*.

### Golimumab

Because GLM was approved only recently (May 2013) by the FDA for treatment of the UC, data are not yet available for this drug in relation to IBD pregnancies.

### Certolizumab pegol

Because CZP lacks the Fc portion of the antibody, which is crucial for the main transfer of immunoglobulins across the placenta, the transfer of CZP to the offspring is low, but a small transfer of Fab fragments is seen [31]. In a group of 10 women receiving CZP during pregnancy, with the final dose 1 to 4 weeks before delivery, CZP was detectable at minimal levels in the infants’ circulation [17]. Overall, CZP is transferred across the placenta to a much lower degree than IFX or ADA [17].

Compared with the other two TNF inhibitors approved for CD, the human data on pregnancy outcomes in mothers treated with CZP during pregnancy are more limited. In three case reports where CZP injections were administered during the third trimester, healthy infants were delivered at full term [84-86], and in a case series of 16 pregnancies of CZP-treated mothers, all children were healthy [31]. In a recent case series with 139 pregnancies, 17 were in mothers with rheumatoid arthritis; of these 139 pregnancies, 103 ended in live birth (2 with congenital abnormalities; see Table 1), 21 in spontaneous miscarriage, and 15 in elective termination [87], results that are similar to those reported in the general population [87]. Based on these findings and the low placental transfer of CZP, the drug appears to be safe to use throughout pregnancy [17]. One of the reports observed uterine bleeding in the mother at 7 days post-partum; whether this can be ascribed to the CZP treatment remains to be established [86].

### Evaluation of the identified studies

In the studies included in this systematic review, more than 1,426 live births occurred among all the women exposed to IFX, ADA, or CZP. The number of congenital abnormalities associated with live births reported did not point to any pattern of specific birth defects (Table 1). There were 128 miscarriages, 81 elected abortions (presumably ‘under-reported’), 12 stillbirths, and 33 preterm births reported (Table 1).

Owing to considerable variation in study designs, selection of controls, and the definition and selection of outcomes in the identified studies, we were unable to conduct a meta-analysis. However, it appears clear from Table 2 that for all outcomes except congenital abnormalities, the OR or RR did not point systematically towards a negative or positive effect of TNF-α inhibitors. A slightly increased risk of congenital abnormalities in women with IBD exposed to TNF-α inhibitors compared with unexposed pregnant women with IBD seemed to be suggested from the few studies with controls (Table 2), but the CIs were very broad. Moreover, this observation should be weighed against the fact that the vast majority of case reports did not report congenital abnormalities, although it is thought that such ‘positive’ cases are more likely to be described and published than negative ones. Lastly, the meta-analysis by Cornish *et al*. [1] including 3,907 women with IBD, showed that an underlying increased risk of the studied outcomes already exists (stillbirths: OR 1.48 (95% CI 0.89 to 2.47); preterm delivery: OR 1.87 (1.52 to 2.31); low birth weight: OR 2.10 (1.38 to 3.19); and congenital abnormalities: OR 2.37 (1.47 to 3.82)) independent of treatment, compared with 320,531 pregnant non-IBD controls.

## Discussion

When summarizing the existing data in this systematic review, no association between treatment with TNF-α inhibitors for IBD in pregnancy and adverse pregnancy outcomes such as spontaneous abortions, preterm deliveries, stillbirth, low birth weight, congenital malformations, and/or infections, was found, even after administration of TNF-α blockers in the third trimester. In studies combining biologics with thiopurines (which often occurs because of the favorable results of the Study of Immunomodulator Naïve Patients in Crohn’s disease (SONIC) [88] and UC SUCCESS (a study similar to the SONIC trial) [89] studies in CD and UC, respectively), there was no increased risk of infections in newborns compared with an unexposed control group.

Ideally, any woman with IBD intending a pregnancy should have counseling with her gastroenterologist and obstetrical provider well in advance of conception, and this counseling should cover the medical treatment options and the risks associated with active disease during pregnancy. Pregnancy should if possible be planned, and the IBD should be controlled before conception. Further, the importance of adherence to treatment and prevention of relapse during pregnancy should be stressed, and the patient should be monitored as a high-risk pregnancy. However, in real-world situations, this is not always the case, but it is important for the physician to discuss with each patient with IBD the risk versus benefit ratio of medical therapy, including TNF-α inhibitors, for disease control during pregnancy.

Embryo-fetal perinatal developmental toxicity studies performed in cynomolgus monkeys receiving doses of TNF-α inhibitors several hundred times the recommended human dose did not reveal any evidence of teratogenic or other deleterious effects [90], such as adverse pregnancy or maternal outcomes [91]. Because IFX does not cross-react with TNF-α in species other than humans and chimpanzees, other animal reproduction studies have not been conducted with this drug. Thus, to date there is no evidence that TNF blockers are associated with embryotoxicity, teratogenicity, or increased pregnancy loss compared with pregnancies unexposed to biologics, either in rheumatology [92], or in a recent retrospective IBD multicenter study [35].

The present systematic literature review reveals that many reports on biologic treatment in pregnancy lack information on the total number of women exposed to treatment, disease activity, co-morbidities, and/or concomitant medications. In some cases, it is therefore difficult to establish whether instances of fetal harm are due to medications or to the severity of the underlying illness [20,21]. Furthermore, many of the studies intending to assess the effect of TNF-α inhibitors *per se* were confounded by the fact that many patients are on multiple medications. Finally, it should be kept in mind that biologics are more commonly used in patients with more severe disease activity, which accordingly might influence the pregnancy outcome data presented [1], especially if patients are not compared with pregnant women with a similar degree of IBD disease severity treated with other medications. As an example, the unfavorable outcome described by Srinivasan [21] may potentially be due to the underlying severity of IBD or to other medications.

The aggravation of underlying disease might be more detrimental to the viability of pregnancy than the apparently low risk of continuing biologic therapies, and the results from this systematic review do not support the practice of stopping biologic treatment of the expectant mother after week 30 because of a theoretical risk of infections or congenital abnormalities in the offspring. The data presented here suggest that the rates of congenital malformations and adverse birth events are similar to the rates in the background population of pregnant women, or at least of women with IBD in general [1]. Further, there is no increased risk of infections in the offspring of mothers on biologic treatment during pregnancy. However, it should be emphasized that immunosuppression of the offspring contraindicate the use of live vaccines until the biologic agent is no longer detectable in the child’s circulation, because of the risk of fatal infections [63,93,94].

### Limitations of the study

Limitations of studies identified in this systematic review included small sample sizes and paucity of studies with control groups. Future studies assessing the outcome of pregnancies exposed to IFX or other biologics during conception and pregnancy should be prospective in nature, and should assess comparable women with IBD with or without the relevant exposure as a control group to draw firm conclusions. Data on potential confounders, especially maternal age, disease activity, and co-medications, should also be recorded. Furthermore, long-term follow-up studies of children exposed to biologic medications in the uterus should be carried out, especially as no long-term data exist on outcomes in children related to the development of diseases such as asthma or autoimmune diseases.

## Conclusions

There is a growing body of evidence suggesting that biologic agents are of low risk in pregnancy. Although it is difficult to prove absolute safety, this systematic review suggests that women who inadvertently become pregnant while taking TNF-α inhibitors can be reassured that continuation of pregnancy does not appear to impose an increased risk to either themselves or their baby. The limited clinical results available suggest that the benefit of biologic agents in attaining response and maintaining remission in pregnant women with IBD might outweigh the risk of pregnancy complications due to flare-ups (for example, spontaneous abortions, prematurity, low birth weight or stillbirth), and the theoretical risk of drug exposure to the fetus [34]. However, the long-term implications of fetal exposure to therapeutic monoclonal antibodies on the child’s developing immune system are still unknown.

Therefore, biologics should be discontinued during pregnancy only if the IBD of the pregnant patient is in remission using stopping criteria for patients with IBD in general, and the intentional use of biologics throughout pregnancy should be considered in situations where the disease is active at the end of the second trimester, as a documented benefit of TNF-α inhibitors for active inflammatory and fistulizing IBD is unprecedented. Further prospective studies are essential to elucidate the risk of combination therapy on the neonatal immune system.

## Abbreviations

ADA: Adalimumab; BCG: Bacillus Calmette-Guérin; CD: Crohn’s disease; CZP: Certolizumab pegol; EMA: European Medicines Agency; FcRn: Fc receptor neonatal; FDA: Food and Drug Administration; GLM: Golimumab; IBD: Inflammatory bowel disease; IFX: Infliximab; MTX: Methotrexate; TNF: Tumor necrosis factor; UC: Ulcerative colitis.

## Competing interests

TJ and OHN have nothing to disclose. EVL has consulted for Abbott Labs, UCB Pharma, and Janssen Biotech, and has received research support from Abbott Labs, UCB Pharma, and Janssen Biotech. However, all authors declare they have not been supported from any organization for the submitted work; have had no financial relationships with any organizations that might have an interest in the submitted work in the previous 3 years; and have no other relationships or activities that could appear to have influenced the submitted work.

## Authors’ contributions

OHN conceived the study; TJ and OHN extracted the data; TJ, OHN, and EVL analysed the data, interpreted the results, and drafted the manuscript; and TJ and OHN extracted the data. EVL is the guarantor. All authors read and approved the final manuscript.

## Pre-publication history

The pre-publication history for this paper can be accessed here:

http://www.biomedcentral.com/1741-7015/11/174/prepub
